# Universal mtDNA fragment for Cervidae barcoding species identification using phylogeny and preliminary analysis of machine learning approach

**DOI:** 10.1038/s41598-023-35637-z

**Published:** 2023-06-05

**Authors:** Ewa Filip, Tomasz Strzała, Edyta Stępień, Danuta Cembrowska-Lech

**Affiliations:** 1 Institute of Biology, University of Szczecin, Wąska 13, 71-415 Szczecin, Poland; 2 The Centre for Molecular Biology and Biotechnology, University of Szczecin, Szczecin, Poland; 3 Department of Genetics, Faculty of Biology and Animal Science, Wrocław University of Environmental and Life Sciences, Wrocław, Poland; 4 Institute of Marine and Environmental Sciences, University of Szczecin, Adama Mickiewicza 16, 70-383 Szczecin, Poland; 5 Sanprobi Sp. z o. o. Sp. k., Kurza Stopka 5C, 70-535 Szczecin, Poland

**Keywords:** Molecular biology, Environmental sciences

## Abstract

The aim of the study was to use total DNA obtained from bone material to identify species of free-living animals based on the analysis of mtDNA fragments by molecular methods using accurate bioinformatics tools Bayesian approach and the machine learning approach. In our research, we present a case study of successful species identification based on degraded samples of bone, with the use of short mtDNA fragments. For better barcoding, we used molecular and bioinformatics methods. We obtained a partial sequence of the mitochondrial cytochrome b (*Cytb*) gene for *Capreolus capreolus*, *Dama dama*, and *Cervus elaphus*, that can be used for species affiliation. The new sequences have been deposited in GenBank, enriching the existing Cervidae mtDNA base. We have also analysed the effect of barcodes on species identification from the perspective of the machine learning approach. Machine learning approaches of BLOG and WEKA were compared with distance-based (TaxonDNA) and tree-based (NJ tree) methods based on the discrimination accuracy of the single barcodes. The results indicated that BLOG and WEKAs SMO classifier and NJ tree performed better than TaxonDNA in discriminating *Cervidae* species, with BLOG and WEKAs SMO classifier performing the best.

## Introduction

The current state of knowledge of molecular biologists has led to the widespread use of mitochondrial DNA (mtDNA) as a marker for species-specific identification in animals^1–5^. For intraspecific detection of unrelated individuals, sequences with high variability are recommended, e.g. certain nuclear genes^6^. For species identification within Cervidae, we choose conservative sequences shared among the animals with species-specific variables, because doing so brings the best effect^7–9^.

Mitochondrial DNA is known to be an effective molecular marker in phylogenetic analyses^10,11^. This is due to the high polymorphism of the control region, as well as a lack of recombination, and very good isolation efficiency, even from small amounts of biological tissue, as well as the resistance of mtDNA to degradation processes. Different gene regions such as mtDNA, have been used for the DNA barcoding approach, but cytochrome c oxidase *COI*) is a universal DNA barcode for animals^12^, such Cervidae^13,14^. Ward et al.^15^ analysed mtDNA *COI* sequences in animals and indicated that the success of barcoding depended upon recent speciation, incorrect morphological taxonomy and species hybridisation, where barcoding could not differentiate interspecies. There are many drawbacks to the use of a barcoding tool for species identification, so, the scientific community must be cautious in accepting the above factors and use additional genes for further clarification. Generally, biological phenomena, such as the hybridisation process of close species, natural introgression process, error in specimen identification using classical taxonomy and recent speciation process, are known to strongly interfere with the DNA barcoding process, and these phenomena are known to occur at different degrees depending on the animal groups and datasets^16–19^. So, it is authoritative to have not only more databases on individual species *COI* gene sequences from different geographical locations and correct identification of species through traditional taxonomy. It should also be noted that taxonomic decisions based on a single molecular marker that is maternally inherited might not resolve all species identification and should be supported by a second molecular marker, such as cytochrome b (*Cytb*).

The analysis of species-specific variation using the homologous cytochrome b (*Cytb*) is characterized by high reproducibility and sensitivity of results^11,20–24^. To distinguish closely related species, selected mtDNA fragments with very high specificity are needed. Often conservative gene sequences encoding proteins are used in studies on interspecies diversity^25^, while the control region is used to provide a reliable source of knowledge about intraspecific variability^7,10,25^.

In some cases, cytochrome b provides excellent phylogenetic information on the taxonomic position of various vertebrates; and thus, it can be used in the analysis of live specimens or for forensic identification purposes^26–29^, with the same success rate as *COI*^30^. In addition, this gene is often considered when determining the origins of samples from difficult biological materials, i.e. hair, feathers, tooth fragments or other bones, which mainly utilize mitochondrial DNA polymorphisms^26^. Irwin et al.^31^ have determined the rate of evolutionary changes for the genera of some species in different components of cytochrome b amino acid sequences based on fossil DNA analyses. Several recent studies show that when the DNA template is derived from bone material, a 300–500 bp *Cytb* fragment, is suitable for mammalian species identification^12,27,32–34^.

Machine learning (ML) is a branch of artificial intelligence (AI) where machines are trained to solve self-designed problems by learning new rules through repeated trials and feedback. ML enables inferring of models or relationships by learning from data. With the development of AI, machine learning has been rapidly developed and applied in DNA barcoding. Over the years, several different analytical methods were devised for the assessment of the species discrimination ability, such as TaxonDNA, NJ tree and machine learning approaches (BLOG and WEKA). Most machine learning approaches (MLA) bear its origin from statistical methods of regression analysis. Machine learning approaches are computer tools, which can be successfully applied in species identification^35^. BLOG (Barcoding with LOGic) and WEKA (Waikato Environment for Knowledge Analysis) are methods of ML, which can recognize unknown species (query set) present in the reference dataset composed of DNA barcode sequence (training set) of known species^36,37^.

Based on the literature, we have found the cytochrome b mitochondrial gene to be useful in identifying species of wild animals using bone material (mandible, frontal bone) and we tested its usefulness on the real life example. The specific aim of our research was to develop a short universal fragment from mtDNA, which could be used in the species identification of various deer populations. In addition, we proposed the use of machine learning approach methods to classify species with DNA barcode sequences.

## Results

### DNA isolation

Table 1 show a spectrophotometer readings on DNA isolates, giving OD 260/280 ratios ranging from 1.8 to 2.33, for the 18 different bone fragments. Among the studied samples, values exceeding 2.0 were obtained for several samples: two fragments of red deer bone marked KBMICSZ3 (2.01), the fallow deer bone fragment KBMICSZ13 (2.21), and a fragment of roe deer frontal bone marked KBMICSZ25 (2.33).

**Table 1:** List of the study materials and results of DNA isolations.

No.	Sample ID	Species	Type of bone	Weight [g]	DNA concentration [ng/μL)]	A260/A280 ratio
1	KBMICSZ1	*Cervus elaphus*	Mandible	0.28	216.8	1.95
2	KBMICSZ2	*Cervus elaphus*	Mandible	0.26	386.0	1.85
3	KBMICSZ3	*Cervus elaphus*	Mandible	0.26	91.1	2.01
4	KBMICSZ9	*Cervus elaphus*	Mandible	0.35	142.8	1.85
5	KBMICSZ20	*Cervus elaphus*	Mandible	0.24	259.8	1.83
6	KBMICSZ4	*Cervus elaphus*	Mandible	0.38	180.2	1.87
7	KBMICSZ5	*Capreolus capreolus*	Frontal bone	0.36	1264.2	1.82
8	KBMICSZ6	*Capreolus capreolus*	Frontal bone	0.34	661.8	1.83
9	KBMICSZ8	*Capreolus capreolus*	Frontal bone	0.28	187.8	1.85
10	KBMICSZ14	*Capreolus capreolus*	Frontal bone	0.35	474.0	1.85
11	KBMICSZ15	*Capreolus capreolus*	Frontal bone	0.36	242.3	1.88
12	KBMICSZ16	*Capreolus capreolus*	Frontal bone	0.39	289.4	1.86
13	KBMICSZ21	*Capreolus capreolus*	Frontal bone	0.30	782.9	1.81
14	KBMICSZ22	*Capreolus capreolus*	Frontal bone	0.30	123.2	1.87
15	KBMICSZ24	*Capreolus capreolus*	Frontal bone	0.30	119.1	1.97
16	KBMICSZ25	*Capreolus capreolus*	Frontal bone	0.30	12.9	2.33
17	KBMICSZ7	*Dama dama*	Mandible	0.33	353.7	1.83
18	KBMICSZ13	*Dama dama*	Mandible	0.28	12.5	2.21

The place of collection samples: ^No. 1–6 and 17–18^ Plecemin: 53°16′31.931″ N 16°48′30.246″ E; ^No. 7–8^ Drawsko Pomorskie 53°31.8336′N 15°48.5802′E; ^No. 9–12^ Stara Korytnica 53°18′2.902″ N 16°2′15.199″ E; ^No. 13–14^ Karwowo 53°41′26.002" N 15°33′2.002″ E; ^No. 15–16^ Dorowo 53°43′20.775″ N 15°27′26.097″ E.

### Species identification of analysed DNA sample

As a result of performing PCR and DNA sequencing on the collected deer samples, 18 sequences of the *Cytb* gene were obtained, which helped in the identification of each species belonging to the Cervidae family. This analysis involved 18 nucleotide sequences with a total of 207 positions in the final dataset. The average GC content was 50%. The *Cytb* region was characterized by a high level of monomorphism with a small number of 163 sites and polymorphic sites a number of 44 and a number of parsimoniously informative sites number (PIC) of 2. Based on the whole length of the *Cytb* gene sequenced, a total of 5 haplotypes were detected with a Hd (Haplotype diversity) equal to 0.771. The most frequent haplotype was Hap_3, which were found among 7 individuals. It should be mentioned that the type of genetic frequency of these haplotypes in North-western Poland Cervidae haplotypes Hap_1: and Hap_2: for *Cervus elaphus*, Hap_3: and Hap_4: for *Capreolus capreolus*, and Hap_5 was for *Dama dama* (Table S3).

**Figure 1:**
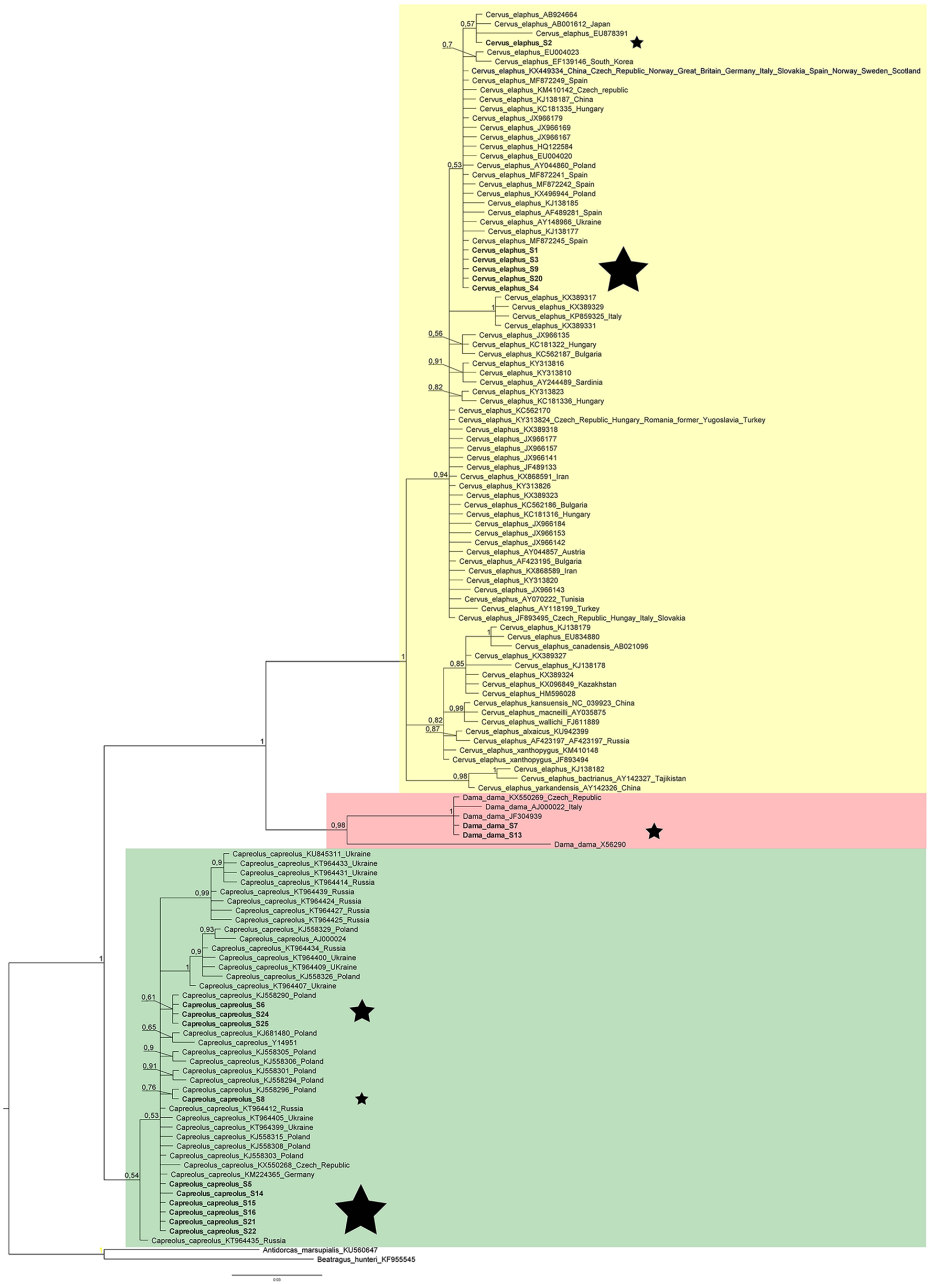
Bayesian phylogenetic tree showing species identification of analysed DNA samples (samples are indicated with a star). Sequences of *Antidorcas marsupialis* and *Beatragus hunteri* were used for rooting. Numbers along nodes are the posterior probability values of nodes. Tree was generated with MrBayes 3.2.6^38^.

Figure 1 show the obtained phylogenetic tree, which was resolved into three distinct clades that consisted of representatives of the three analysed species. Samples were grouped together with each species representative showing a high probability (100%) of assignment, indicating clear species identification. Within the clades, we found substantial polytomy, which is a result of a lack of sequence informativity within the species level.

**Figure 2:**
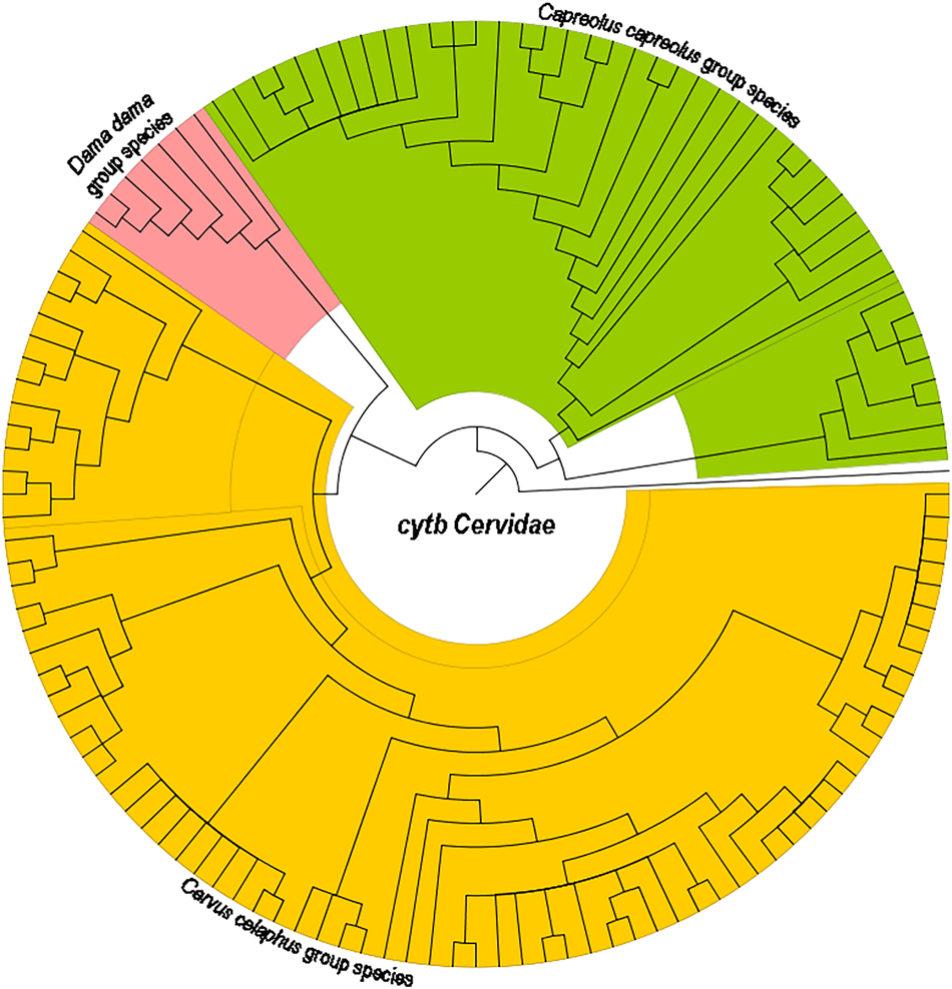
Phylogenetic tree inferred from the *Cytb* sequences. Results from the ML and the MP analyses were mapped onto the NJ tree. Tree was generated with FigTree 1.4.4^39^.

Another phylogenetic analysis inferred from the *Cytb* sequences was constructed to illustrate the phylogenetic relationship of Cervidae species based on *Cytb* sequences from GenBank and our studies (Fig. 2). A phylogenetic ML tree was constructed using a pre-trained neural network. The tree important Cervidae species were formed monophyletic clades and found well-supported with bootstrap values (> 80%).

Machine learning approaches of BLOG and WEKA were compared with distance-based (TaxonDNA) and tree-based (NJ tree) methods based on discrimination accuracy and cost-effectiveness of barcode gene (Table 2, Fig. 2). The results indicated that BLOG and WEKAs SMO classifier and NJ tree performed better than TaxonDNA in discriminating Cervidae species. Specifically, the single barcode *Cytb* exhibited the highest species resolution (100%) for identifying 3 Cervidae species when BLOG or WEKAs SMO classifiers were used. This study showed that machine learning approaches provided higher discrimination accuracy and cost-effectiveness over other analytical methods in DNA barcoding of Cervidae species.

**Table 2:** Species resolution success rates for the *Cervidae* based on different analytical methods.

Rates, %	BLOG	WEKA				TaxonDNA	NJ tree
		Naïve Bayes	SMO	Jrip	J48		
Correctly identify	100.00	60.00	100.00	94.00	94.00	90.00	100.00
Misidentify	0.00	40.00	0.00	6.00	6.00	10.00	0.00
Not identify	0.00	0.00	0.00	0.00	0.00	0.00	0.00

**Figure 3:**
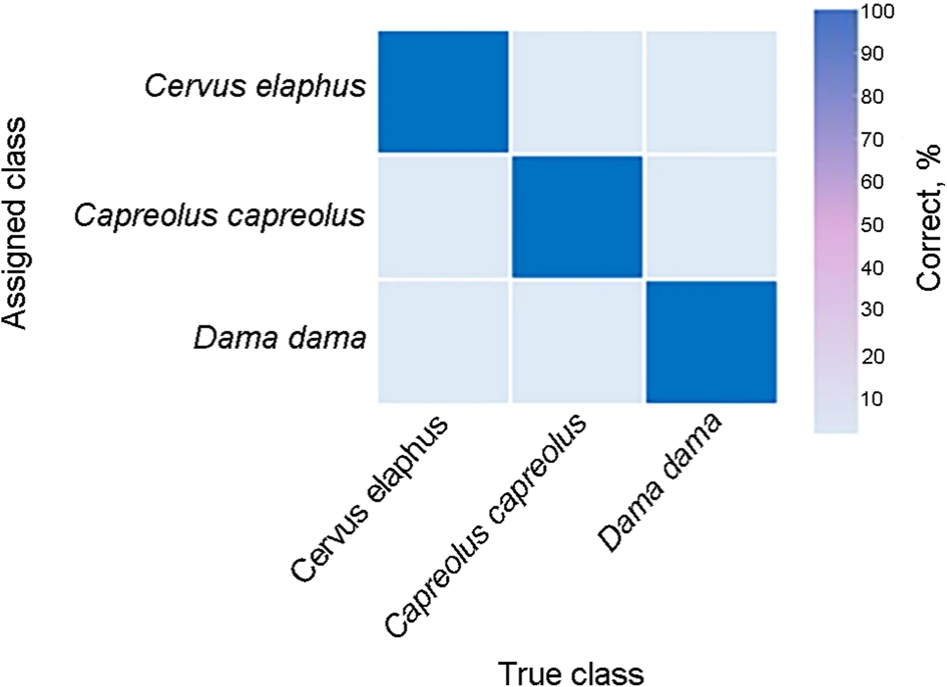
Confusion matrix of WEKAs SMO classifier generated by cytb showing classification results of Cervidae species, obtained with R environment^40^.

Moreover, cytb region also provided the highest accuracy of species discrimination (100%) when using WEKAs SMO classifier (Fig. 3).

## Discussion

We present that DNA barcoding is an effective molecular tool for Cervidae species identification and phylogenetic inferences as a result of our research, we have obtained the new sequences that have been deposited in a section of the Genbank belonging to the Cervidae family. Identification of species must be effective and precise even from degraded environmental material. In addition, well-resolved molecular phylogenies derived from these DNA barcode sequences have the potential to improve investigations of the mechanisms underlying community assembly and functional trait evolution. Our proposed methodology can be used in the future as a routine marker in cases when degraded samples will be used. It also contributes to the development of the subject of species identification in different environments. With the development of DNA barcoding, several analytical methods were developed for the assessment of species discrimination ability. There are no criteria for evaluating the quality of the analytical methods for species discrimination. We have analyzed the effect of barcodes on species identification from the perspective of the machine learning approach (MLA). We tested a reinforcement-learning algorithm to solve the challenge of reconstructing phylogenetic trees, which are used to describe the relationships among a set of sequences. Current tools for phylogenetic- tree reconstruction integrate heuristic approaches to evaluate only a subset of all potential trees, thus they suffer from the known trade-off between accuracy and running time. In our study, we tested the methodology for predicting the maximum likelihood tree. Our preliminary results, based on a machine learning approach algorithm demonstrate that the trained algorithm can accurately and efficiently reconstruct maximum-likelihood trees. This development technique ML could provide rapid, simple, and reliable tools for species confirmation and can be applied to the modelling of species distribution.

In the present study, we proposed and tested the use of a relatively short mitochondrial DNA sequence for the species identification of members of the genus Cervidae. Our proposed methodology, based on machine learning, confirmed the identification results of the classical phylogeny-based approach, which will enable its wider use in future routine studies of this type.

Nowadays, DNA analysis of biological samples has become the standard practice in animal identification at various taxonomic levels. Different types of tissues, such as bones, blood, hair (fur), feathers, skin, meat (muscle sample), faecal, and others are often the subject of many studies in various DNA analysis laboratories^22,41^. A universal fragment of genetic information is constantly being sought to use in many areas, e.g. poaching^26,42,43^, illicit trafficking of endangered species^26,42–44^, protection of endangered animal species^27^, or determination of meat origin (for identification purposes)^44,45^. Anna Ramón-Laca et al.^22^, showed that differentiation of species can be achieved by using a species-specific primer that amplifies dissimilar length fragments. There are differences of opinion among the researchers, regarding which of the markers, *COI* or *Cytb*, provides more reliable and reproducible results for DNA barcoding analysis. In 2010, a group of researchers led by Tobe et al.^1^ carried out an assessment of genetic intraspecific variability based on *COI* and *Cytb* sequences from 217 mammalian species. The results showed that the discriminatory power was higher for the *Cytb* gene, i.e. there was a higher probability that two random individuals from a given population would have sequence differences at the marker locus than for the *COI* sequence. Research carried out by Wilson-Wilde et al.^46^ demonstrated that identification based on the *COI* gene sequence is suitable for genetically distant species, while in the case of closely related species, it is no longer unambiguous and requires additional tests. However, *COI*, *Cytb*, and the mt-CR control region are still used for this purpose^1,20–24,47–50^. To find better molecular tools the compilation of known DNA markers led to the construction of the genetic map of *Cervus elaphus*^51–53^. This genetic map comprises 621 sites (length of 2532 cM, with average intervals of 5.7 cM), and it integrates modern technologies and research methods, including comparative genomics and orthologous alleles of DNA markers derived from ruminants and other mammals (i.e. Pere David’s deer, *Elaphurus davidianus* and red deer, *C. elaphus*)^54^. The genetic map of deer was used as an annotation for further research, such as the origin and evolution of ruminant genomes^52^, QTL scanning^53^, SNP analyses of the whole genome^55,56^ and whole genome sequencing as well as the annotation and assembly of pseudochromosomes^54^.

In the results presented in this study, the total length of DNA fragments of all analysed individuals was 207 bp due to the removal of the last nucleotides in the sequences. The reason for obtaining different lengths was probably due to the inhibition of sequencing reactions by individual matrices. Similar results were obtained by Gupta et al.^33^, who worked on stool samples and also obtained short *Cytb* sequence fragments of 366, 374 and 503 bp^22,41,57^. We show that when using bone tissue, the primers used in this work for the *Cytb* gene fragment amplifications work better because firstly, they differentiate closely related species well and have the additional advantage that they can be used for many other mammalian species as well. Our research is confirmed by many studies, not only for the family Cervidae but also other works on the identification of other wild mammalian species^1,5,32,41,48,58^. Our phylogenetic analysis grouped the analysed sequences within individual species with 100% probability (Fig. 1). The *Cytb* fragment, analysed in this study, allows correct species identification, however, the lack of intraspecific polymorphism results in the inability to use it in population studies. This is clearly shown in the phylogenetic tree obtained (Fig. 1) where most monospecific nodes are polytomous. The lack of the node’s solution (polytomy) is in this case is the result of a lack of genetic information from the analysed DNA sequences (soft polytomy). Our results suggest that the intraspecific genetic polymorphism is low for all mammalian species. Similar results were obtained in earlier studies^1,59^.

MLAs extract the distinct features from the DNA sequences by training the reference dataset and then used for identifying the query sequences. Cytb region provided the highest species resolution when using the BLOG method, and WEKAs SMO classifier. In the comparison of TaxonDNA and NJ tree, the BLOG and SMO methods produced a relatively low level of misidentification. Moreover, though the above methods have to achieved > 90% species identification success rate, still there is a need for further improvement in success rate (Fig. 2, Table 2). The discrimination ability of combined different barcodes in the species of the Cervidae genus is still fully unknown.

We present that DNA barcoding is an effective molecular tool for *Cervidae* species identification and phylogenetic inferences a result of our research. Identification of species must be effective and precise even from degraded environmental material. In addition, well-resolved molecular phylogenies derived from these DNA barcode sequences have the potential to improve investigations of the mechanisms underlying community assembly and functional trait evolution. Our proposed methodology can be used in the future as a routine marker in cases when degraded samples will be used. It also contributes to the development of the subject of species identification in different environments.

With the development of DNA barcoding, several analytical methods were developed for the assessment of species discrimination ability. There are no criteria for evaluating the quality of the analytical methods for species discrimination. We have analyzed the effect of barcodes on species identification from the perspective of the machine learning approach (MLA). We tested a reinforcement-learning algorithm to solve the challenge of reconstructing phylogenetic trees, which are used to describe the relationships among a set of sequences. Current tools for phylogenetic- tree reconstruction integrate heuristic approaches to evaluate only a subset of all potential trees, thus they suffer from the known trade-off between accuracy and running time. In our study, we tested the methodology for predicting the maximum likelihood tree. Our preliminary results, based on a machine learning approach algorithm demonstrate that the trained algorithm can accurately and efficiently reconstruct maximum-likelihood trees. This development technique ML could provide rapid, simple, and reliable tools for species confirmation and can be applied to the modelling of species distribution.

## Materials and methods

### Sampling DNA

In total 18 skull samples were obtained from wild-living specimens of three ungulate species in 2016-2018. DNA isolations were performed using the column-based method and the GeneMatrix Bond DNA Purification Kit (Eurx). The purity and concentration of DNA from the bone material were determined using a NanoDrop 2000c spectrophotometer (Thermo Scientific) (Table 1).

### Mitochondrial DNA analysis

The following primer pair was used for PCR amplification^32^: Mcb_KPF398: TACCATGAGGACAAATATCATTCTG, Mcb_KPR869:CCTCCTAGTTTGTTAGGGATTGATCG.

PCR reactions were performed in a total volume of 20 μL consisting of 20 ng of DNA, 1× DreamTaq Buffer with MgCl_2_, 0.2 mM dNTP, 0.2 μM of each primer, and 1 U DreamTaq DNA Polymerase (Thermo Scientific). The thermal reaction profile used to amplify the *Cytb* regions was as follows: initial denaturation at 95 °C for 2 min followed by 35 cycles of denaturation at 95 °C for 30 s, annealing at 58 °C for 30 s, extension of the primer at 72 °C for 30 s, and a final extension of 72 °C for 7 min.

PCR products were checked by electrophoresis in a 1.5% agarose gel containing ethidium bromide and a TBE buffer (pH 8.0); the gels were visualized under UV and archived using the GeneSys V.1.3.5.0 software (Syngene). The sequences reported in this paper have been deposited in the GenBank nucleotide sequence database with the accession numbers marked '*' in Tables S1, S2.

### Sequence analysis

At first, the forward and reverse sequences were aligned, and consensus sequences were obtained using Basic Local Alignment Tool software. ClustalW and Mega7.1 software were used to perform multiple sequence alignments^60^. Substitution patterns and rates were estimated under the Kimura 2-parameter model^61^.

The genetic variability of haplotypes was characterized by the total alignment length (bp), the number of monomorphic sites, the number of polymorphic sites, the number of parsimony informative sites (PIC), the number of haplotypes, and the average G + C content in each region using DnaSP6.10.01^62^.

### Species identification

To reveal the species of each sample analysed, we performed phylogeny reconstruction using the Bayesian approach. Seven *Cytb* sequences for *Cervus elaphus*, two for *Dama dama* and 9 for *Capreolus capreolus* were grouped together along with 131 *Cytb* sequences (Tables S1, S2) of the three species from Genbank, as well as two outgroup sequences (*Antidorcas marsupialis*, *Beatragus hunteri*) for comparison. Next, all sequences were aligned with the Muscle algorithm^61^ and cut to obtain the proper alignment set in Seaview^63^. The best-fit substitution model was chosen using jModelTest 2.10^64^. Finally, the tree was constructed with MrBayes 3.2.6^38^ using two, randomly started and independent runs, carried out for 20,000,000 generations of Markov chain steps. A consensus tree was constructed based on the set of trees collected after both runs converged—i.e. when the standard deviation of both runs was much below 0.01. ML tree was also searched using DeepNNPhylogeny, pre-trained neural networks to predict the best models of sequence evolution and the best tree topologies^65^. All neural networks have been trained with a large number of alignments simulated with the software PolyMoSim, designed to test phylogenetic tree reconstruction and to train machine learning models for phylogenetic reconstruction. We used also ModelTeller, a machine-learning based algorithm, which is based on the Random Forest, for the prediction of the optimal phylogenetic model for branch-length estimation^66^.

### The MLAs BLOG and WEKA (machine learning approach)

BLOG 2.0 (Barcoding with LOGic; Institute of Systems Analysis and Computer Science, National Research Council, Rome, Italy) and WEKA (The University of Waikato, Hamilton, New Zealand) were applied. BLOG provides a supervised MLA, which selects suitable nucleotide positions and computes the logic formulae for species identification^36^. The WEKA workbench is used for classification, clustering and selection problems^37,67^. The four classifiers: Naïve Bayes^68^, support vector machines (SMO)^69^, the decision tree C4.5 (J48)^70^ and the rule-based RIPPER (Jrip)^71^ were implemented to analyse the DNA sequences.

### Distance-based analysis (TaxonDNA)

The Kimura^72^ 2-parameter model (K2P)distances between all sequence pairs were calculated with TaxonDNA 1.9 (National University of Singapore, Singapore) and applied to compute the mean and the range of the intra- and interspecific distances for the barcode. The relative distribution of the pairwise intra- and inter-specific distances were estimated with the “best match” and “best close match” functions in the TaxonDNA under the Kimura^72^ 2-parameter (K2P) distance model.

### Tree-based analysis (neighbour-joining)

The phylogenetic analysis was carried out in the MEGA11 (Center for Evolutionary Medicine and Informatics, The Biodesign Institute, Tempe, AZ, USA) based on the K2P model with 1000 bootstrap replications and pairwise deletions^73^. Species discrimination was considered successful only when all the conspecific individuals formed a monophyletic clade.

### Ethics approval and consent to participate

Under Poland law, Institutional Animal Ethics Committee approval was not required for the study of Cervidae.

### Statement

The consent of the bioethical commission is not required for this type of research in Poland. This is due to the fact that the study material was not taken from live animals, but from bones. In addition, the animals were not caught, euthanized or killed. Samples were taken from fallen animals by North West Forest Districts and sent for this study.

## Conclusions

Despite the challenging biological material of bone tissue, the Cytb gene was successfully used to identify individuals of closely related ungulate bone DNA species using PCR analysis, Sanger DNA sequencing and accurate bioinformatics tools such as the Bayesian approach and the machine learning approach. This research will be extended to analyse more sequences of many barcodes for Cervidae species identification using DNA barcode sequences through machine learning approaches. The data obtained will serve used for comparisons with gene bank records. The proposed methodology will be helpful as a routine identification procedure for a variety of tissue sources, even in cases where the samples are degraded.

## Limitations

The efficiency of the applied DNA isolation method varied. The resulting DNA concentration values demonstrated over a 100-fold difference between the lowest and highest concentration. The study revealed that one of the most important moments during the DNA extraction process was the preliminary preparation of the bone material. Identification of bone samples depended on the quality and quantity of DNA present in the sample. The efficiency of the applied DNA isolation method varied. The resulting DNA concentration values demonstrated over a 100-fold difference between the lowest and highest concentration. The study revealed that one of the most important moments during the DNA extraction process was the preliminary preparation of the bone material. Identification of bone samples depended on the quality and quantity of DNA present in the sample.

## Supplementary Information

Supplementary Table S1.

Supplementary Table S2.

Supplementary Table S3.

## Data Availability

All data generated or analyzed during this study are included in this published article. The datasets generated during and/or analysed during the current study are available from the corresponding author on reasonable. All the sequences have been deposited in NCBI GenBank and can be found under accession numbers No: MK575604-MK575603. The *Cytb* sequences are deposited on the corresponding author’s data.
