# Supplementary material for: Universal mtDNA fragment for Cervidae barcoding species identification using phylogeny and preliminary analysis of machine learning approach
Source: Sci Rep. 2023 Jun 5;13:9133. doi: 10.1038/s41598-023-35637-z (PMC10241948; doi:10.1038/s41598-023-35637-z)
Supplement: Supplementary file 1 — Supplementary Table S1. [file 41598_2023_35637_MOESM1_ESM.docx]

Universal mtDNA fragment for Cervidae barcoding species identification using phylogeny and preliminary analysis of machine learning approach

Ewa Filip ^1,2*^, Tomasz Strzała ^3^, Edyta Stępień ^4^ and Danuta Cembrowska-Lech ^1,5^

^1^ Institute of Biology, University of Szczecin, Wąska 13, 71-415 Szczecin, Poland; ewa.filip@usz.edu.pl ORCID: 0000-0003-2313-8398; danuta.cembrowska-lech@usz.edu.pl ORCID: 0000-0002-1503-0064

^2^ The Centre for Molecular Biology and Biotechnology, University of Szczecin, Poland; ewa.filip@usz.edu.pl ORCID: 0000-0003-2313-8398

^3^ Department of Genetics, Faculty of Biology and Animal Science, Wrocław University of Environmental and Life Sciences, Wrocław, Poland; tomasz.strzala@upwr.edu.pl ORCID: 0000-0002-7761-1630

^4^ Institute of Marine and Environmental Sciences, University of Szczecin, Adama Mickiewicza 16, 70-383 Szczecin, Poland; edyta.stepien@usz.edu.pl ORCID: 0000-0002-5638-7676 5

^5^ Sanprobi Sp. z o. o. Sp. k., Kurza Stopka 5c, 70-535 Szczecin, Poland; danuta.cembrowska@sanprobi.pl ORCID: 0000-0002-1503-0064

* Correspondence: ewa.filip@usz.edu.pl

Supplementary Information

Table S1. The *Cytb* sequences were obtained from Genbank and used in phylogenetic reconstruction.

| Species | Accession number in GenBank/*Sequences number in this work | Species | Accession number in GenBank/*Sequences number in this work |
| --- | --- | --- | --- |
| *Cervus elaphus* | KX449334 | *Cervus elaphus* | KX389327 |
| *Cervus elaphus* | MF872249 | *Cervus elaphus* | KJ138178 |
| *Cervus elaphus* | KM410142 | *Cervus elaphus* | KX389324 |
| *Cervus elaphus* | KJ138187 | *Cervus elaphus* | KX096849 |
| *Cervus elaphus* | KC181335 | *Cervus elaphus* | HM596028 |
| *Cervus elaphus* | JX966179 | *Cervus elaphus* | KJ138179 |
| *Cervus elaphus* | JX966169 | *Cervus elaphus* | KJ138182 |
| *Cervus elaphus* | JX966167 | *Cervus elaphus* | EU834880 |
| *Cervus elaphus* | HQ122584 | *Cervus elaphus alxaicus* | KU942399 |
| *Cervus elaphus* | EU004023 | *Cervus elaphus xanthopygus* | KM410148 |
| *Cervus elaphus* | EU004020 | *Cervus elaphus kansuensis* | NC39923 |
| *Cervus elaphus* | AY044860 | *Cervus elaphus xanthopygus* | JF893494 |
| *Cervus elaphus* | AB924664 | *Cervus elaphus macneilli* | AY035875 |
| *Cervus elaphus* | MF872241 | *Cervus elaphus wallichi* | FJ611889 |
| *Cervus elaphus* | MF872242 | *Cervus elaphus canadensis* | AB021096 |
| *Cervus elaphus* | KX496944 | *Cervus elaphus bactrianus* | JF893495 |
| *Cervus elaphus* | KC562170 | *Cervus elaphus yarkandensis* | AY142327 |
| *Cervus elaphus* | KJ138185 | *Cervus elaphus* | AY142326 |
| *Cervus elaphus* | EF139146 | *Dama dama* | KX550269 |
| *Cervus elaphus* | AF489281 | *Dama dama* | AJ000022 |
| *Cervus elaphus* | AY148966 | *Dama dama* | KX550269 |
| *Cervus elaphus* | AB001612 | *Dama dama* | AJ000022 |
| *Cervus elaphus* | KY313824 | *Dama dama* | JF304939 |
| *Cervus elaphus* | KX389318 | *Dama dama* | X56290 |
| *Cervus elaphus* | KJ138177 | *Dama dama* | *MK575604 |
| *Cervus elaphus* | JX966177 | *Dama dama* | *MK575605 |
| *Cervus elaphus* | JX966157 | *Capreolus capreolus* | KT964412 |
| *Cervus elaphus* | JX966141 | *Capreolus capreolus* | KT964405 |
| *Cervus elaphus* | JX966135 | *Capreolus capreolus* | KT964399 |
| *Cervus elaphus* | JF489133 | *Capreolus capreolus* | KJ681480 |
| *Cervus elaphus* | KX868591 | *Capreolus capreolus* | KJ558315 |
| *Cervus elaphus* | KY313826 | *Capreolus capreolus* | KJ558308 |
| *Cervus elaphus* | KY313823 | *Capreolus capreolus* | KJ558305 |
| *Cervus elaphus* | KX389323 | *Capreolus capreolus* | KJ558303 |
| *Cervus elaphus* | KC562186 | *Capreolus capreolus* | KJ558301 |
