# Supplementary material for: Universal mtDNA fragment for Cervidae barcoding species identification using phylogeny and preliminary analysis of machine learning approach
Source: Sci Rep. 2023 Jun 5;13:9133. doi: 10.1038/s41598-023-35637-z (PMC10241948; doi:10.1038/s41598-023-35637-z)
Supplement: Supplementary file 2 — Supplementary Table S2. [file 41598_2023_35637_MOESM2_ESM.docx]

Universal mtDNA fragment for Cervidae barcoding species identification using phylogeny and preliminary analysis of machine learning approach

Ewa Filip ^1,2*^, Tomasz Strzała ^3^, Edyta Stępień ^4^ and Danuta Cembrowska-Lech ^1,5^

^1^ Institute of Biology, University of Szczecin, Wąska 13, 71-415 Szczecin, Poland; ewa.filip@usz.edu.pl ORCID: 0000-0003-2313-8398; danuta.cembrowska-lech@usz.edu.pl ORCID: 0000-0002-1503-0064

^2^ The Centre for Molecular Biology and Biotechnology, University of Szczecin, Poland; ewa.filip@usz.edu.pl ORCID: 0000-0003-2313-8398

^3^ Department of Genetics, Faculty of Biology and Animal Science, Wrocław University of Environmental and Life Sciences, Wrocław, Poland; tomasz.strzala@upwr.edu.pl ORCID: 0000-0002-7761-1630

^4^ Institute of Marine and Environmental Sciences, University of Szczecin, Adama Mickiewicza 16, 70-383 Szczecin, Poland; edyta.stepien@usz.edu.pl ORCID: 0000-0002-5638-7676 5

^5^ Sanprobi Sp. z o. o. Sp. k., Kurza Stopka 5c, 70-535 Szczecin, Poland; danuta.cembrowska@sanprobi.pl ORCID: 0000-0002-1503-0064

* Correspondence: ewa.filip@usz.edu.pl

**Supplementary Information**

Table S2. The *Cytb* sequences were obtained from Genbank and used in phylogenetic reconstruction.

| Species | Accession number in GenBank/*Sequences number in this work | Species | Accession number in GenBank/*Sequences number in this work |
| --- | --- | --- | --- |
| *Cervus elaphus* | KC181322 | *Capreolus capreolus* | KJ558296 |
| *Cervus elaphus* | KC181316 | *Capreolus capreolus* | KJ558290 |
| *Cervus elaphus* | JX966184 | *Capreolus capreolus* | KX550268 |
| *Cervus elaphus* | JX966153 | *Capreolus capreolus* | *MK575596 |
| *Cervus elaphus* | JX966142 | *Capreolus capreolus* | *MK575597 |
| *Cervus elaphus* | AY044857 | *Capreolus capreolus* | *MK575606 |
| *Cervus elaphus* | AF423195 | *Capreolus capreolus* | *MK575598 |
| *Cervus elaphus* | KX868589 | *Capreolus capreolus* | *MK575599 |
| *Cervus elaphus* | KY313820 | *Capreolus capreolus* | *MK575600 |
| *Cervus elaphus* | KY313816 | *Capreolus capreolus* | *MK575601 |
| *Cervus elaphus* | KC562187 | *Capreolus capreolus* | *MK575602 |
| *Cervus elaphus* | KC181336 | *Capreolus capreolus* | *MK575603 |
| *Cervus elaphus* | JX966143 | *Capreolus capreolus* | KJ558294 |
| *Cervus elaphus* | *MK575589 | *Capreolus capreolus* | KT964435 |
| *Cervus elaphus* | *MK575590 | *Capreolus capreolus* | Y14951 |
| *Cervus elaphus* | *MK575591 | *Capreolus capreolus* | KT964407 |
| *Cervus elaphus* | *MK575592 | *Capreolus capreolus* | KT964434 |
| *Cervus elaphus* | *MK575593 | *Capreolus capreolus* | KT964400 |
| *Cervus elaphus* | *MK575594 | *Capreolus capreolus* | KJ558329 |
| *Cervus elaphus* | *MK575595 | *Capreolus capreolus* | KT964409 |
| *Cervus elaphus* | AY070222 | *Capreolus capreolus* | KT964439 |
| *Cervus elaphus* | KY313810 | *Capreolus capreolus* | KJ558326 |
| *Cervus elaphus* | AY118199 | *Capreolus capreolus* | KT964424 |
| *Cervus elaphus* | AY244489 | *Capreolus capreolus* | AJ000024 |
| *Cervus elaphus* | MF872245 | *Capreolus capreolus* | KT964433 |
| *Cervus elaphus* | KX389317 | *Capreolus capreolus* | KT964431 |
| *Cervus elaphus* | EU878391 | *Capreolus capreolus* | KT964427 |
| *Cervus elaphus* | KX389329 | *Capreolus capreolus* | KT964425 |
| *Cervus elaphus* | KP859325 | *Capreolus capreolus* | KT964414 |
| *Cervus elaphus* | KX389331 | *Capreolus capreolus* | KM224365 |
| *Cervus elaphus* | AF423197 |  |  |
