# Supplementary material for: Universal mtDNA fragment for Cervidae barcoding species identification using phylogeny and preliminary analysis of machine learning approach
Source: Sci Rep. 2023 Jun 5;13:9133. doi: 10.1038/s41598-023-35637-z (PMC10241948; doi:10.1038/s41598-023-35637-z)
Supplement: Supplementary file 3 — Supplementary Table S3. [file 41598_2023_35637_MOESM3_ESM.docx]

Universal mtDNA fragment for Cervidae barcoding species identification using phylogeny and preliminary analysis of machine learning approach

Ewa Filip ^1,2*^, Tomasz Strzała ^3^, Edyta Stępień ^4^ and Danuta Cembrowska-Lech ^1,5^

^1^ Institute of Biology, University of Szczecin, Wąska 13, 71-415 Szczecin, Poland; ewa.filip@usz.edu.pl ORCID: 0000-0003-2313-8398; danuta.cembrowska-lech@usz.edu.pl ORCID: 0000-0002-1503-0064

^2^ The Centre for Molecular Biology and Biotechnology, University of Szczecin, Poland; ewa.filip@usz.edu.pl ORCID: 0000-0003-2313-8398

^3^ Department of Genetics, Faculty of Biology and Animal Science, Wrocław University of Environmental and Life Sciences, Wrocław, Poland; tomasz.strzala@upwr.edu.pl ORCID: 0000-0002-7761-1630

^4^ Institute of Marine and Environmental Sciences, University of Szczecin, Adama Mickiewicza 16, 70-383 Szczecin, Poland; edyta.stepien@usz.edu.pl ORCID: 0000-0002-5638-7676 5

^5^ Sanprobi Sp. z o. o. Sp. k., Kurza Stopka 5c, 70-535 Szczecin, Poland; danuta.cembrowska@sanprobi.pl ORCID: 0000-0002-1503-0064

* Correspondence: ewa.filip@usz.edu.pl

**Supplementary information**

**Table S3**. Mitochondrial DNA cytochrome *b* (Cyt*b*) respective haplotypes found in the *Cervidae* populations in Poland, their frequencies in the whole sample and the GenBank accession numbers for these haplotypes.

| Sample ID | Species | *Cytb*- mtDNA haplotype frequency (%) | Type of Haplotype | Sequence accession no. |
| --- | --- | --- | --- | --- |
|  |  |  |  | *Cytb* |
| KBMICSZ1 | *Cervus elaphus* | 5.6 | Hap_1 | MK575589 |
| KBMICSZ2 | *Cervus elaphus* | 1.1 | Hap_2 | MK575590 |
| KBMICSZ3 | *Cervus elaphus* | 5.6 | Hap_1 | MK575591 |
| KBMICSZ9 | *Cervus elaphus* | 5.6 | Hap_1 | MK575592 |
| KBMICSZ20 | *Cervus elaphus* | 5.6 | Hap_1 | MK575593 |
| KBMICSZ4 | *Cervus elaphus* | 5.6 | Hap_1 | MK575594 |
| KBMICSZ5 | *Capreolus capreolus* | 7.8 | Hap_3 | MK575595 |
| KBMICSZ6 | *Capreolus capreolus* | 3.3 | Hap_4 | MK575596 |
| KBMICSZ8 | *Capreolus capreolus* | 7.8 | Hap_3 | MK575597 |
| KBMICSZ14 | *Capreolus capreolus* | 7.8 | Hap_3 | MK575606 |
| KBMICSZ15 | *Capreolus capreolus* | 7.8 | Hap_3 | MK575598 |
| KBMICSZ16 | *Capreolus capreolus* | 7.8 | Hap_3 | MK575599 |
| KBMICSZ21 | *Capreolus capreolus* | 7.8 | Hap_3 | MK575600 |
| KBMICSZ22 | *Capreolus capreolus* | 7.8 | Hap_3 | MK575601 |
| KBMICSZ24 | *Capreolus capreolus* | 3.3 | Hap_4 | MK575602 |
| KBMICSZ25 | *Capreolus capreolus* | 3.3 | Hap_4 | MK575603 |
| KBMICSZ7 | *Dama dama* | 2.2 | Hap_5 | MK575604 |
| KBMICSZ13 | *Dama dama* | 2.2 | Hap_5 | MK575605 |
